# Tolerability of MenACWY-TT vaccination in adolescents in the Netherlands; a cross-sectional study

**DOI:** 10.1186/s12889-021-11767-9

**Published:** 2021-09-26

**Authors:** J. M. Kemmeren, L. van Balveren, A. Kant, H. de Melker

**Affiliations:** 1 Center for Infectious Disease Control, National Institute for Public Health and the Environment, PO Box 1, 3720 BA Bilthoven, The Netherlands; 2 Netherlands Pharmocovigilance Centre Lareb, Goudsbloemvallei 7, 5237 MH ‘s-Hertogenbosch, The Netherlands

**Keywords:** MenACWY-TT vaccine, Adolescents, Tolerability, Reactogenicity, Local reactions, Systemic events

## Abstract

**Background:**

In 2018, meningococcal ACWY-TT vaccine (MenACWY-TT) was offered to adolescents in the Netherlands within the National Immunization Programme at 14 years of age. A questionnaire study assessed the tolerability of this vaccine.

**Methods:**

Five thousand adolescents were invited to participate and to fill in two questionnaires about systemic events in the week before vaccination and local reactions and systemic events in the week after vaccination. Frequencies of local and systemic adverse events in the week after vaccination were calculated. Association between the occurrence of systemic symptoms in the week before and after the vaccination was tested by using generalized mixed models (GLMM).

**Results:**

Of all adolescents, 139 returned one or both questionnaires. Any local reaction within 7 days after vaccination was reported by 55.6% of the adolescents. Pain (50%) and reduced use of the injected arm (21.3%) were most often reported. Any systemic event was reported by 67.6% of the participants, with myalgia as the most often reported event (37.0%). Compared with the week before vaccination, there were no increased odds of experiencing systemic symptoms in the week after vaccination (OR 0.95; 95%CI 0.40–2.27).

**Conclusions:**

After vaccination with MenACWY-TT vaccine, most adolescents reported one or more adverse events, which were mostly mild and transient. Systemic symptoms were not reported more often in the week after compared to the week before vaccination. Unfortunately, due to a low response rate we were not able to detect the absolute elevated risks the sample size calculation was based on. However, despite limited data, our results are in line with results from prelicensure data, and indicate that MenACWY-TT vaccination is well tolerated in adolescents.

**Supplementary Information:**

The online version contains supplementary material available at 10.1186/s12889-021-11767-9.

## Background

Invasive meningococcal disease (IMD) affects individuals of all ages, with the highest incidence observed in young children and a second peak in incidence in adolescents and young adults. Since 2015 the incidence of IMD caused by capsular group W in the Netherlands has been increasing [1]. Mortality also increased, including infant and adolescent deaths.

Vaccination is the best option to prevent IMD. The endemic increase in serogroup W disease has led to changes in the National Immunization Program (NIP) in the Netherlands [1]. In the spring of 2018, the single-dose quadrivalent ACWY-TT (MenACWY-TT) vaccine replaced meningococcal C vaccination in toddlers of 14 months of age. Furthermore, in the autumn of 2018 a new vaccination moment was introduced whereby MenACWY-TT vaccine was offered to adolescents of 14 years of age. In the spring of 2019, a catch-up MenACWY-TT vaccination was offered to adolescents aged 15–18 years old.

The MenACWY-TT vaccine included in the NIP was licensed in the Netherlands in 2012 [2]. Several clinical trials showed that this vaccine is safe and well tolerated in toddlers, adolescents and young adults [3–15]. Pain was the most common local event and fatigue and headache were the most commonly reported systemic events [16]. Most reactogenicity events were mild to moderate in intensity, of short duration, and resolved spontaneously.

According to the WHO’s advice [17], it is in the Netherlands the policy that after major changes in the NIP, the profile of frequently occurring AEs after vaccinations will be assessed. In addition, since the age-range of participants in the clinical trials is wide (i.e. 11 to 25 years), the objective of this study was to evaluate the tolerability of a single dose of MenACWY-TT in adolescents in the Netherlands, in the new target population of adolescents of 14 years of age. In a comparable tolerability study after introduction of the vaccination against human papilloma virus (HPV), a high proportion of short-term adverse events was found [18]. Improved knowledge of the occurrence of adverse events, including mild and transient symptoms whether or not causally related to the vaccination, allows adolescents and parents to have correct information and expectations. This will help prevent possible vaccine refusal.

## Methods

### Study population and setting

In this observational study, adolescents were asked to fill in questionnaires about complaints they experienced in the week before and in the weeks after the MenACWY vaccination. The study population consisted of a group of healthy adolescents around the age of 14 year, who were vaccinated with MenACWY-TT vaccine, according to the NIP schedule in the Netherlands (see Table 1). Five thousand adolescents eligible for this vaccine, were randomly selected from the vaccination register Praeventis of the National Institute for Public Health and the Environment, the RIVM. In Praeventis, all children under the age of 19 years, residing in the Personal Records Database (PRD) in the Netherlands and eligible for the NIP are registered. Through a link with the PRD, Praeventis receives continuous updates on all newborn and deceased children and on changes in the address of children (due to movement within the country or immigration/emigration). Praeventis provides a crucial tool for the evaluation of the NIP by for example conducting specific studies where individuals included in the immunisation register are approached for further research.

**Table 1:** Characteristics of the responders before and after vaccination

Characteristics		Total population (***n*** = 181) n (%)	Before vaccination (***n*** = 139) n (%)	After vaccination (***n*** = 108) n (%)
**Sex**	M	88 (48.6)	69 (49.6)	54 (50.0)
	F	93 (51.4)	70 (50.4)	54 (50.0)
**Chronic Illness**	No		80 (57.6)	
	Yes		59 (42.4)	
**Medication use**	No		82 (59.0)	
	Yes		34 (24.5)	
	Unknown		23 (16.5)	
**Medical intervention**	No		111 (79.9)	
	Yes		5 (3.6)	
	Unknown		23 (16.5)	
**Other complaints**	No		108 (77.7)	
	Yes		8 (5.8)	
	Unknown		23 (16.5)	
**Ill on day immunization**	No			104 (96.3)
	Yes			4 (3.7)

All adolescents were selected from sites that organized mass vaccinations in October and November 2018. Both adolescents and their parents received an invitation letter to participate in the study. In the letter to the adolescents, a flyer with information concerning the study was included. The adolescents could register on a website as participant by creating an account until 1 day before vaccination. During the registration process, the planned date of vaccination and informed consent from the adolescent and their parents was required. If the registration was done within 4 days before the scheduled vaccination, the first questionnaire was available immediately. Otherwise, the adolescent received an email invitation to access the first questionnaire within their account on the website. The invitation to fill in the second questionnaire was sent 7 days after vaccination. In this questionnaire, the vaccination date was verified. A reminder was sent after 2 days and the adolescent could fill in the questionnaire until day 11 after vaccination.

The first questionnaire was about symptoms that may have occurred in the week prior to vaccination, the second was about symptoms observed within 1 week after vaccination to obtain information about adverse events (AEs) of the MenACWY-TT vaccine (local reactions and systemic events). A third questionnaire was sent 14 days after vaccination only to those who had reported that one or more adverse events were not recovered after filling in the second questionnaire. Similarly, a fourth questionnaire was send 28 days after vaccination to those who had reported that one or more adverse events were not recovered after filling in the third questionnaire.

### Vaccines

The MenACWY-TT vaccine (Nimenrix®, Pfizer) was given intramuscularly in the non-dominant arm. The 0.5-ml dose contained *Neisseria meningitidis*-group A polysaccharide 5 μg, *Neisseria meningitidis*-group C polysacharide 5 μg, *Neisseria meningitidis*-group W-135 polysacharide 5 μg, and *Neisseria meningitidis*-group Y polysacharide 5 μg, conjugated to tetanus toxoid carried protein 44 μg.

### Questionnaires

The first questionnaire included questions about the onset of systemic events in the week before vaccination (see supplementary file ‘Questionnaire before vaccination’). Systemic events included addressed fatigue, sleeping problems, being irritable, fever, cough, symptoms of common cold, being listless/apathetic, having dyspnea, headache, decreased appetite, vomiting, diarrhea, lower abdominal pain, nausea, dizziness, fainting, myalgia, joint pain, muscular spasm, sweating, rash, itch, and other complaints. The presence was dichotomized (yes/no). Additionally, the use of analgesics, occurrence of medical intervention, absence from school, sport and/or other activities, and parents’ or guardians’ absence from work were asked.

The second questionnaire about AEs within 1 week after the vaccination included the same questions as in the first questionnaire, supplemented with questions about the occurrence of local reactions (see supplementary file ‘Questionnaire 1 week after vaccination’). Local reactions included swelling, redness, and pain at the injection site (see Table 2 for severity grading scales [19]). Also the course of each symptom was asked: time to onset, outcome and duration. In the third and fourth questionnaire the outcome and duration of not recovered adverse events were asked (see supplementary files ‘Questionnaire 2 weeks after vaccination’ and ‘Questionnaire 4 weeks after vaccination’).

**Table 2:** Frequency of swelling and/or redness within 7 days after vaccination (*n* = 108)

	Swelling	Redness	Pain	Swelling armpit	Reduced use of arm
Severity (n; %)					
None	101 (93.5)	104 (96.3)	54 (50.0)	105 (97.2)	85 (78.7)
< 2.5 cm / mild	7 (6.5)	3 (2.8)	32 (29.6)	1 (0.9)	15 (13.9)
2.5–5 cm / moderate	0 (−)	1 (0.9)	19 (17.6)	2 (1.9)	5 (4.6)
> 5 cm / pronounced	0 (−)	0 (−)	3 (2.8)	0 (−)	3 (2.8)
Recovered (% yes)	7 (100)	4 (100)	52 (96.3)	3 (100)	23 (100)
Mean onset time in hr. (sd; range)	1.1 (1.1; 0.05–3.0)	1.3 (1.4; 0.03–3.0)	2.0 (5.5; 0.0–24)	18.3 (9.8; 7.0–24)	7.1 (10.3; 0.0–24)
Mean duration in hr. (sd; range)	40.1 (38.5; 5.0–120)	42.1 (35.8; 0.5–72)	55.6 (35.9; 0.0–144)	56.0 (13.9; 48–72)	55.0 (32.2; 0.0–120)

### Statistical analysis

Assuming a percentage of fever of 2.1% (95% CI 1.0–4.3) in a comparative group of adolescents, i.e. girls of 12 years old before HPV vaccination (personal communication), a 95% confidence level and a width of the confidence interval of 5%, the sample size should be 324 adolescents to be able to detect an absolute elevated risk of 2.5% [20]. Assuming a response rate of 10% and a drop-out rate of 30%, 5000 adolescents needed to be invited for participation.

The percentage of adolescents experiencing AEs within 1 week after vaccination and 95% CI were computed by type and severity of the AE. The association between the occurrence of systemic symptoms in the week before and the week after the vaccination were analysed by means of a Generalized Linear Mixed model (GLMM) to address dependency of data. Proportions of absence from school, sport and/or other activities, parents’ absence from work and medical intervention within 7 days after vaccination were calculated with a 95% CI, as well as the association of these items before and after vaccination by GLMM. All GLMM analyses were adjusted for sex.

In the second questionnaire, the answer category ‘unknown’ for the presence of a symptom was included, while this was not included in the first questionnaire. To determine whether this has affected the results, a sensitivity analysis was performed by including these participants either in the category with the symptom and in the category without the symptom.

Analyses were performed using SPPS statistics 24 and SAS version 9.4.

## Results

### Response rate and population characteristics

In total, 5000 invitations letters were sent to adolescents who were eligible for the MenACWY-TT vaccination and their parents. Of them, 181 agreed to participate (3.6%). The response rate for the questionnaire about symptoms in the week before vaccination was 76.8% (*n* = 139/181). For the questionnaire about symptoms within 7 days after vaccination, the response rate was 59.7% (*n* = 108). Both questionnaires were returned by 104 adolescents. A third and fourth questionnaire was sent to 28 and 11 adolescents, respectively. Of these, 20 (71.4%) completed the third and 6 (54.5%) completed the fourth questionnaire.

Of all respondents, 50.4% were male in the week before the vaccination, and 50.0% in the week after the vaccination (see Table 1). About 43% of all adolescents reported on having a chronic illness, with eczema (*n* = 29), allergies (*n* = 25), hay fever (*n* = 17) and asthma (*n* = 16) as most reported illness.

### Local reactions after MenACWY-TT vaccination

Table 2 shows the frequency, severity, onset time and duration of local reactions that occurred within 7 days after vaccination.

One or more local reactions within 7 days after vaccination were reported by 60 adolescents (55.6%) with a total of 92 local reactions reported. Pain and reduced use of the arm of the injection site were reported most as local reaction. Almost all reactions recovered within 7 days after vaccination (*n* = 260/262; 99.2%), with two adolescents who reported in the second questionnaire that injection site pain had not yet ceased to exist. Upon the third questionnaire 14 days after vaccination, both adolescents reported that the injection site pain had disappeared within 1 day after the onset. The mean time of onset of the different local reactions was within 18 h after vaccination. The mean duration ranged from 40.1–56.0 h.

### Systemic AEs after MenACWY-TT vaccination

One or more systemic events within 1 week after vaccination were reported by 67.6% of the participants. A total of 221 systemic AEs were reported. Of these symptoms, myalgia was the systemic event most often reported (37.0%) with a mean duration of 2.4 days. Colds (22.2%) and headache (22.2%) were then most reported (see Table 3). The mean onset time of systemic AEs was 31 h after vaccination. Most of the systemic AEs (75.5%) recovered within 7 days with a mean duration of 64 h (i.e. 2.7 days) (see Table 3). Eighteen participants (8.1%) reported in the third questionnaire that they had recovered after a mean duration of 157 h (i.e. 6.5 days). Another 8 participants (3.6%) reported in the fourth questionnaire (sent after 28 days) to be recovered with a mean duration of 17 days. Two patients with fatigue and sleep problems reported in the fourth questionnaire not to be recovered as yet.

**Table 3:** Reported frequency of systemic events within 7 days after immunisation (*n* = 108)

	n (%)	Recovered n (%)	Mean onset time hr. (sd; range)	Mean recover time hr. (sd; range)
Fatigue	19 (17.6)	10 (52.6)	19.9 (18.0; 0.50–72.0)	66.0 (56.4; 12.0–216)
Sleeping problems	4 (3.7)	1 (25.0)	25.0 (33.0; 0.00–72.0)	72.0 (−)
Irritable	7 (6.5)	4 (57.1)	18.0 (26.1; 0.00–72.0)	72.0 (48.0; 48.0–144)
Fever	3 (2.8)	3 (100)	32.0 (36.7; 0.00–72.0)	48.0 (41.6; 24.0–96.0)
Cough	10 (9.3)	5 (50.0)	31.2 (27.8; 0.00–96.0)	96.0 (37.9; 48.0–144)
Common cold	24 (22.2)	13 (54.2)	41.2 (41.4; 0.00–144)	81.2 (46.5; 24.0–144)
Listless/apathetic	8 (7.4)	6 (75.0)	21.8 (14.2;3.0–48.0)	72.0 (30.4; 48.0–120)
Dyspnea	5 (4.6)	4 (80.0)	28.8 (20.1; 0.01–48.0)	36.8 (45.2; 0.07–96.0)
Headache	24 (22.2)	22 (91.7)	30.7 (27.7;0.00–96.0)	51.9 (54.0; 0.0–144)
Decreased appetite	7 (6.5)	3 (42.9)	55.3 (54.4; 0.00–120)	33.3 (25.4; 4.0–48.0)
Vomiting	1 (0.9)	0 (0)	216 (−)	–
Diarrhea	5 (4.6)	4 (80.0)	15.8 (20.4;0.00–48.0)	18.0 (12.0; 0.00–24.0)
Lower abdominal pain	11 (10.2)	10 (90.9)	49.9 (46.1 (0.00–144)	68.4 (60.4; 12.0–216)
Nausea	8 (7.4)	8 (100)	20.8 (32.0; 0.00–96.0)	52.0 (45.8; 1.0–144)
Dizziness	10 (9.3)	6 (60.0)	36.2 (43.7; 0.00–144)	60.2 (60.0; 1.0–168)
Fainting	0	–	–	–
Myalgia	40 (37.0)	40 (100)	12.3 (30.8; 0.00–192)	56.8 (44.1; 0.00–240)
Joint pain	5 (4.6)	4 (80.0)	38.8 (43.2; 0.00–96.0)	96.0 (43.8; 48.0–144)
Muscular spasm	9 (8.3)	8 (88.9)	45.9 (65.8; 0.03–192)	73.8 (71.3; 2.00–240)
Sweating	2 (1.9)	2 (100)	24.0 (0.00; 24.0–24.0)	96.0 (67.9; 48.0–144)
Rash	7 (6.5)	4 (57.1)	68.6 (72.6; 0.00–168)	37.0 (56.3; 0.00–120)
Itch	7 (6.5)	7 (100)	41.5 (49.1; 0.00–120)	61.7 (49.7; 24.0–144)
Other	5 (4.6)	3 (60.0)	24.0 (17.0; 0.02–48.0)	19.9 (34.5; 4.0–72.0)

Adolescents with local reactions, reported more often systemic AEs (84.7%) than adolescents without local reactions (51.1%) (*p* < 0.001). This pattern was particularly seen for myalgia (*p* < 0.001), dyspnea (*p* = 0.044) and listless (*p* = 0.044).

Adolescents with chronic illness reported significantly more on dizziness after vaccination than adolescents without chronic illness (OR 1.28, 95% CI 1.27–34.6). No other significant between adverse events and chronic illness were found (data not shown).

### Symptoms before and after MenACWY-TT vaccination

No increased odds on prevalence of systemic symptoms were reported after vaccination compared with the week before vaccination (Table 4).

**Table 4:** Odds of systemic adverse events within 1 week after MenACWY vaccination adjusted for the odds before vaccination

	OR (95% CI)^a^
Fatigue	0.34 (0.18–0.65)
Sleeping problems	0.22 (0.07–0.67)
Irritable	0.29 (0.12–0.71)
Fever	1.01 (0.22–4.71)
Cough	0.51 (0.22–1.17)
Common cold	0.50 (0.27–0.91)
Listless/apathetic	0.24 (0.10–0.55)
Dyspnea	0.81 (0.25–2.65)
Headache	0.70 (0.38–1.29)
Decreased appetite	0.55 (0.21–1.45)
Vomiting	0.20 (0.02–1.77)
Diarrhea	0.57 (0.19–1.73)
Lower abdominal pain	0.57 (0.26–1.25)
Nausea	0.66 (0.27–1.66)
Dizziness	0.73 (0.31–1.71)
Fainting	Did not converge
Myalgia	1.04 (0.60–1.78)
Joint pain	0.44 (0.15–1.27)
Muscular spasm	1.75 (0.61–5.01)
Sweating	Did not converge
Rash	0.39 (0.16–0.99)
Itch	0.43 (0.17–1.07)
Other	0.81 (0.25–2.61)
All systemic events	0.43 (0.23–0.81)

^a^GLMM analysis; adjusted for sex

A sensitivity analysis was done where participants who filled in ‘unknown’ for having a symptom after vaccination were included in the category of adolescents with the symptom (‘yes’) or in the category of adolescents without the symptom (‘no’), respectively. Including the unknowns in the ‘no’ category did not change the results. For fever (OR 2.70; 95% CI 0.78–9.37) and dyspnea (OR 1.65; 95%CI 0.61–4.46) higher odds were found when the unknowns were included in the ‘yes’ category, although both associations were still non-significant. The significant association of irritability (OR 0.62; 95%CI 0.30–1.26) and rash (OR 0.49; 95%CI 0.21–1.17) after vaccination disappeared when the unknowns were included in the ‘yes’ category.

### Absence and medical intervention

Absence from school, sport and/or other activities within 7 days after vaccination was reported in 6.5% of the adolescents. The median duration was 1 day. However, higher frequencies of absence were found in the week before vaccination. When adjusted for the frequencies found in the week before vaccination, no differences were found in absence from school and sport activities after vaccination (see Table 5). None of the parents or guardians were absent from work to take care of the vaccinated child.

**Table 5:** Frequencies and odds ratios in absence and medical intervention before (*n* = 139) and after (*n* = 108) MenACWY vaccination

	Before vaccination (%; 95% CI))	After vaccination (%; 95% CI))	OR (95% CI)^**a**^
Absence from school	9.4 (4.2–14.6)	4.6 (0.2–9.1)	0.49 (0.16–1.48)
Absence from sport	8.6 (3.6–13.7)	4.6 (0.2–9.1)	0.55 (0.18–1.66)
Absence from other activities	0 (−0.4–0.4)	0 (−0.5–0.5)	Did not converge
Analgesic use	24.5 (17.0–32.0)	12.0 (5.4–18.6)	0.43 (0.21–0.91)
Care from parents/guardians	0.7 (−1.1–2.5)	0 (−0.5–0.5)	Did not converge
Medical intervention	3.6 (0.1–7.1)	0 (−0.5–0.5)	Did not converge

^a^GLMM analysis; adjusted for sex

Analgesics within 7 days after vaccination were used by 12.0% of the adolescents. All of them used paracetamol and the median duration was 1 day. The frequency of analgesic use was statistically significant lower in the week after vaccination compared to the week before vaccination (see Table 5).

None of the adolescents needed medical intervention in the week after vaccination.

## Discussion

One pillar in the safety surveillance of vaccines is to examine the tolerability of newly introduced vaccines. Therefore, the present study was undertaken to assess the tolerability of meningococcal ACWY vaccine in healthy adolescents. The results showed that adverse events after vaccination were frequently experienced, and pain in the arm of injection and myalgia were the adverse events most often reported. The frequency of systemic events in the week after vaccination was not higher compared with the week before vaccination. This gives context that, although known AEs, at least a part of these complaints may be not related to the vaccination. The reported adverse events were mostly mild and transient. Only two adolescents reported not to be recovered from their symptoms after 28 days. However, both reported to suffer already from these systemic symptoms in the week before the vaccination.

The frequencies of AEs after vaccination found in our study are comparable with the results of previous studies with vaccines of the same composition. Ostergaard et al. also found that pain was the most common local reaction and fatigue and headache the most common systemic event [3]. In other phase 2 studies similar findings were reported. All studies concluded that MenACWY-TT vaccine had an acceptable safety and reactogenicity profile [5, 13, 21, 22], with reactogenicity events which were mostly mild to moderate in intensity, of short duration and which resolved spontaneously. The Summary of Product Characteristics of Nimenrix® also describes a similar patterns of adverse events [2], although in our study fever was less frequently reported, probably due to differences in age distribution of the study populations.

Several factors may bias the results in observational studies, such as in our study. First, it was not possible to include an unvaccinated control group. Therefore, we collected data about systemic symptoms occurring in the week prior to the vaccination and the week after vaccination in order to provide context of the results.

Secondly, the response rate in this study was very low. In the sample size calculation we used response and dropout rates from previous studies of adverse events after vaccination. In the end, the response and failure rates in the present study turned out to be much lower. One reason may be the complicated registration method due to the new privacy legislation (since 2018 both the teenager and the parents have to give digital permission). A second reason could be related to the target group and their interests. Possibly a lot of eligible participants were not interested in the study. In the future, methods will be sought to make the registration procedure and declaration of consent for participation in such a study as simple as possible. The way of communicating with the target group will also have to be critically examined. Perhaps we will have to interview adolescents to determine in what way participating in research is attractive for them.

A low response rate can give rise to sampling bias if the nonresponse is unequal among the participants regarding exposure and/or outcome. It is not clear to what extent this has occurred in this study. Another reason for the low response may be that some adolescents possibly did not participate because they did not experience (severe) adverse reactions after the vaccination. This could result in an overestimation of the frequency of AEs. Conversely, adolescents may not have completed the questionnaire due to the experience of a serious AE. This would result in a underestimation of the number of AEs. However, it seems plausible that people are more motivated to participate if they experience an AE. So the latter effect is likely to be less than non-response due to the absence of AEs.

It is known that the response rate in public health studies is higher among females, older individuals and people with higher education level [23]. In our study, the participants were of the same age, but education level is unknown. However, sex was equally distributed among the respondents. Another characteristic of respondents to web surveys is that they show more social engagement, and are probably more interested in research compared to non-responders [24]. This may suggest that responders will complete the questionnaire truthfully and that despite the low response rate the results of the present study possibly are still accurate.

Next to low participation rate, the dropout within our study was 22.3% (i.e. 104 from 139 adolescents completed the first and second questionnaires). No significant differences in comorbidity and systemic events in the week before vaccination were found between complete responders and the dropouts. So, we have no indication that adolescents who dropped-out compromised a specific subgroup that experienced either fewer or more adverse events.

Because of the low response rate the statistical power of the data collected is low. The sample size was calculated such that an elevated fever risk of 2.5% could be detected. However, because of the small sample size we were able to detect only an elevation of 5.9% or higher. Since the proportion of fever cases before and after vaccination was 2.9 and 2.8% respectively, no significant difference could be detected, even not in the sensitivity analyses where adolescents who answered ‘I don’t know’ for fever after vaccination were recoded as having fever (*n* = 5).

## Conclusions

After vaccination with MenACWY-TT vaccine, the adolescents participated in our study reported particularly pain at the injection site and myalgia. Adverse events were mostly mild and transient. The reported frequency of systemic events and medical care seeking in the week after vaccination is not higher than in the week before vaccination. So, it seems that at least a part of these reported adverse events after vaccination may be not related to the vaccination, although it is hard to draw firm conclusions. Because of the low response rate, we were not able to detect the absolute elevated risks the sample size calculation was based on. However, in line with results from pre-licensure data, our study shows that MenACWY-TT vaccination is well tolerated in adolescents.

## Supplementary Information

**Additional file 1.** Questionnaire about complaints that may occur in the week before the MenACWY vaccination.

**Additional file 2.** Questionnaire about complaints that occur in the week after the MenACWY vaccination.

**Additional file 3.** Questionnaire about unrecovered complaints 2 weeks after the MenACWY vaccination.

**Additional file 4.** Questionnaire about unrecovered complaints 4 weeks after the MenACWY vaccination.

## Data Availability

The datasets used and/or analysed during the current study are available from the corresponding author on reasonable request.

## Abbreviations

AEs: Adverse events
GLMM: Generalized mixed models
HPV: Human papilloma virus
IMD: Invasive meningococcal disease
MenACWY-TT: Meningococcal ACWY vaccine
NIP: National Immunization Programme
RIVM: National Institute for Public Health and the Environment
