# Supplementary material for: Tolerability of MenACWY-TT vaccination in adolescents in the Netherlands; a cross-sectional study
Source: BMC Public Health. 2021 Sep 26;21:1752. doi: 10.1186/s12889-021-11767-9 (PMC8474790; doi:10.1186/s12889-021-11767-9)
Supplement: Supplementary file 1 — Additional file 1. Questionnaire about complaints that may occur in the week before the MenACWY vaccination. [file 12889_2021_11767_MOESM1_ESM.docx]

**Questionnaire about complaints that may occur in the week before the MenACWY vaccination**

In a few days you will receive the vaccination that protects against four types of meningococcal disease (MenACWY). The questionnaire below is about complaints that may occur within one week prior to this vaccination. Filling in this list will take a 5-10 minutes.

**Notes for completing the questionnaire:**

Different types of questions are asked in the questionnaire. For questions with square boxes you can give multiple answers, in a round box only one answer is possible.

When you have answered the questions, you can click on 'send'. After you have submitted the answers, it is no longer possible to change the answers.

**1) What is your gender?**

○ Male

○ Female

**2) What is your birth date? (dd.mm.jjjj)**

**3) What is your height? (in cm)**

**…..**

**4) What is your weight? (in kg)**

**…...**

**5) What is your postal code? (9999)**

**…..**

**6) On what date will you be vaccinated? (dd.mm.jjjj)**

**7) Have you been ill or did you have a cold in the past week?**

○ No

○ Yes, I had a cold

○ Yes, I have had the flu

○ Yes, other namely …..

**8) Has a doctor or general practitioner diagnosed you with one of the following conditions in the past (you can click multiple answers)?**

☐ No, none of the conditions mentioned below

☐ Eczema

☐ Hay fever

☐ Allergy

☐ Asthma

☐ Diabetes

☐ Migraine

☐ Other conditions, namely …..

**9) Have you experienced one or more of the following complaints in the past week?**

No Yes

Listlessness/apathetic ○ ○

Common cold ○ ○

Fever ○ ○ (fill in question 9a)

Headache ○ ○ (fill in question 9c – 9g)

Flu ○ ○

Cough ○ ○

Dyspnea ○ ○

Fatigue ○ ○

Sleeping problems ○ ○

Irritable ○ ○

**9a) How did you measure the temperature?**

○ I did not measure the temperature

○ Rectally *(fill in question 9b)*

○ In the ear *(fill in question 9b)*

○ Other, namely: ….. *(fill in question 9b)*

**9b) What was the maximum value of the temperature?**

…….. ⁰C

**9c) How long did the headache last (if you have had a headache several times, please indicate how long it lasted the most times)?**

○ Less than 1 hour

○ Between 1-4 hours

○ Between 4-24 hours

○ 1 day

○ More than 1 day

**9d) How would you describe the headache?**

○ A pressing/clamping pain

○ A dull pain all over the head

○ A throbbing pain

**9e) Did you experience the headache on one side of the head**

○ yes

○ no

**9f) During the headache, did you feel nauseous or did you vomit?**

○ yes

○ no

**9g) Have you had similar headaches (attacks) before?**

○ yes, it started at the age of └─┴─┘ years and └─┴─┘ months

○ no

**10) Did you experience one of the following complaints in the past week?**

No Yes

Decreased appetite ○ ○

Nausea ○ ○

Vomiting ○ ○

Diarrhea ○ ○

Lower abdominal pain ○ ○

Dizziness ○ ○

Fainting ○ ○

**11) And did you experience one of the following complaints in the past week?**

No Yes

Myalgia ○ ○

Joint pain ○ ○

Muscular spasm ○ ○

Transpire ○ ○

Rash ○ ○

Itch ○ ○

**12) Have you had any other complaint(s) in the past week that have not been mentioned above?**

○ No

○ Yes *(fill in question 12a)*

**12a) What other complaint(s) did you experience?**

**………**

***Complete questions 13 to 16 only if you reported a complaint in the past week***

**13) Have you been reported absent from school, sports and/or other activities in the past week related to the complaints that have occurred?**

No Yes

School ○ ○ (fill in question 13a)

Sport ○ ○ (fill in question 13b)

Other activities ○ ○ (fill in question 13c and d)

**13a) How long have you been absent from school?**

○ less than 1 day

○ 1 day

○ 2 days

○ 3 days

○ 4 days

○ 5 days

○ 6 days

○ 7 days

**13b) How long have you not been exercising?**

○ less than 1 day

○ 1 day

○ 2 days

○ 3 days

○ 4 days

○ 5 days

○ 6 days

○ 7 days

**13c) What kind of activities were you unable to do?**

……

**13d) How long were you unable to these activities?**

○ less than 1 day

○ 1 day

○ 2 days

○ 3 days

○ 4 days

○ 5 days

○ 6 days

○ 7 days

**14) Have your parents or someone else taken time off from work in the past week to take care of you related to the complaints you experienced?**

○ No

○ Yes *(fill in question 14a)*

**14a) How long did he/she take time off from work?**

…. (in hours or days)

**15) Did you take analgesics or other medication in the past week related to the complaints you experienced?**

○ No

○ Yes *(fill in question 15a)*

**15a) Which medicine(s) did you use to treat these complaints?**

**…….**

**16) Did you need medical help in the past week related to the complaints you experienced?**

○ No

○ Yes *(fill in question 16a and 16b)*

**16a) What kind of medical help has been sought related to the complaints you experienced?**

□ Contact youth health care organization by phone

□ Contact general practitioner by phone

□ Visit general practitioner

□ Visit First Aid in hospital

□ Visit medical doctor in hospital

□ Admission to hospital

□ Other, namely: **…….**

**16b) Can you describe the complaints for which you sought medical help?**

…..

***This is the end of this questionnaire. Thank you for your participation!***

Send
