# Supplementary material for: Tolerability of MenACWY-TT vaccination in adolescents in the Netherlands; a cross-sectional study
Source: BMC Public Health. 2021 Sep 26;21:1752. doi: 10.1186/s12889-021-11767-9 (PMC8474790; doi:10.1186/s12889-021-11767-9)
Supplement: Supplementary file 2 — Additional file 2. Questionnaire about complaints that occur in the week after the MenACWY vaccination. [file 12889_2021_11767_MOESM2_ESM.docx]

**Questionnaire about complaints that occur in the week after the MenACWY vaccination**

Last week you received the vaccination that protects against four types of meningococcal disease (MenACWY). The questionnaire below is about complaints that may occur within one week after the vaccination. Filling in this list takes approximately 10 to 15 minutes.

**Notes for completing the questionnaire:**

Different types of questions are asked in the questionnaire. For questions with square boxes you can give multiple answers, in a round box only one answer is possible.

When you have answered the questions, you can click on 'send'.

**1) On what date have you been vaccinated? (dd.mm.jjjj)**

**2) In which arm was the vaccination administered?**

○ Left arm

○ Right arm

**3) Were you ill at the time of vaccination?**

○ No

○ Yes, I had a cold

○ Yes, I had the flu

○ Yes, I had a stomach flu

○ Yes, other namely …..

**The following questions are related to complaints around the injection site within one week after the vaccination.**

**4) Did you experience redness and/or swelling around the injection site?**

No Yes

Swelling ○ ○ *(fill in question 4a to 4d)*

Redness ○ ○ *(fill in question 4e to 4h)*

**4a) Can you indicate the size of the swelling?**

○ less than 2,5 cm

○ between 2,5 and 5 cm

○ greater than 5 cm

**4b) Can you indicate how long after the vaccination the swelling occurred?**

…. (dropdown list minutes/hours/days)

**4c) Have you recovered from the swelling?**

○ No

○ Yes *(fill in question 4d)*

**4d) How long did it take to recover from the swelling?**

…. (dropdown list minutes/hours/days)

**4e) Can you indicate the size of the redness**

○ less than 2,5 cm

○ between 2,5 and 5 cm

○ greater than 5 cm

**4f) Can you indicate how long after the vaccination the redness occurred?**

…. (dropdown list minutes/hours/days)

**4g) Have you recovered from the redness?**

○ No

○ Yes *(fill in question 4h)*

**4h) How long did it take to recover from the redness?**

…. (dropdown list minutes/hours/days)

**5) Did you experience one of the following reactions:**

No yes

Pain at the injection site ○ ○ *(fill in question 5a to 5d)*

Swelling of the armpit ○ ○ *(fill in question 5e to 5h)*

Reduced use of the arm ○ ○ *(fill in question 5i to 5l)*

**5a) What was the severity of the pain around the injection site?**

○ Mild

○ Moderate

○ Pronounced

**5b) Can you indicate how long after the vaccination the pain started?**

…. (dropdown list seconds/minutes/hours/days)

**5c) Have you recovered from the pain?**

○ No

○ Yes *(fill in question 5d)*

**5d) How long did it take to recover from the pain?**

…. (dropdown list seconds/minutes/hours/days)

**5e) What was the severity of the swelling of the armpit?**

○ Mild

○ Moderate

○ Pronounced

**5f) Can you indicate how long after the vaccination the swelling of the armpit occurred?**

…. (dropdown list seconds/minutes/hours/days)

**5g) Is the swelling of the armpit recovered?**

○ No

○ Yes (*fill in question 5h)*

**5h) How long did it take to recover from the swelling of the armpit?**

…. (dropdown list seconds/minutes/hours/days)

**5i) What was the severity o which you could use your arm less?**

○ Mild

○ Moderate

○ Pronounced

**5j) How long after the vaccination did you start using your arm less?**

…. (dropdown list seconds/minutes/hours/days)

**5k) Can you use your arm normally again?**

○ No

○ Yes *(fill in question 5l)*

**5l) How long did it take that you could use your arm normally again?**

…. (dropdown list seconds/minutes/hours/days)

**6) Have you experienced one or more of the following complaints within one week after the vaccination?**

No Yes Do not know

Listlessness/apathetic ○ ○ (fill in question 6a to 6c) ○

Common cold ○ ○ (fill in question 6d to 6f) ○

Fever ○ ○ (fill in question 6g to 6k) ○

Headache ○ ○ (fill in question 6l to 6s) ○

Flu ○ ○ (fill in question 6t to 6v) ○

Cough ○ ○ (fill in question 6w to 6y) ○

Dyspnea ○ ○ (fill in question 6z to 6ab) ○

Fatigue ○ ○ (fill in question 6ac to 6ae) ○

Sleeping problems ○ ○ (fill in question 6af to 6ah) ○

Irritable ○ ○ (fill in question 6ai to 6ak) ○

**6a) Can you indicate how long after the vaccination you started to feel listlessness?**

…. (dropdown list seconds/minutes/hours/days)

**6b) Have you recovered from the listlessness?**

○ No

○ Yes *(fill in question 6c)*

**6c) How long did it take to recover from the listlessness?**

…. (dropdown list seconds/minutes/hours/days)

**6d) Can you indicate how long after the vaccination you caught a cold?**

…. (dropdown list seconds/minutes/hours/days)

**6e) Have you recovered from the cold?**

○ No

○ Yes *(fill in question 6f)*

**6f) How long did it take to recover from the cold?**

…. (dropdown list seconds/minutes/hours/days)

**6g) Can you indicate how long after the vaccination the fever started?**

…. (dropdown list seconds/minutes/hours/days)

**6h) How did you measure the temperature?**

○ I did not measure the temperature

○ Rectally *(fill in question 6i)*

○ In the ear *(fill in question 6i)*

○ Other, namely: ….. *(fill in question 6i)*

**6i) What was the maximum value of the temperature?**

…….. ⁰C

**6j) Have you recovered from the fever**

○ No

○ Yes *(fill in question 6k)*

**6k) How long did it take to recover from the fever?**

…. (dropdown list seconds/minutes/hours/days)

**6l) Can you indicate how long after the vaccination the headache started?**

…. (dropdown list seconds/minutes/hours/days)

**6m) How long did the headache last (if you have had a headache several times, please indicate how long it lasted the most times)?**

○ Less than 1 hour

○ Between 1-4 hours

○ Between 4-24 hours

○ 1 day

○ More than 1 day

**6n) How would you describe the headache?**

○ A pressing/clamping pain

○ A dull pain all over the head

○ A throbbing pain

**6o) Did you experience the headache on one side of the head**

○ yes

○ no

**6p) During the headache, did you feel nauseous or vomit?**

○ yes

○ no

**6q) Have you had similar headaches (attacks) before?**

○ yes, it started at the age of └─┴─┘ years and └─┴─┘ months

○ no

**6r) Have you recovered from the headache**

○ No

○ Yes *(fill in question 6s)*

**6s) How long did it take to recover from the headache?**

…. (dropdown list seconds/minutes/hours/days)

**6t) Can you indicate how long after the vaccination the flu started?**

…. (dropdown list seconds/minutes/hours/days)

**6u) Have you recovered from the flu?**

○ No

○ Yes *(fill in question 6v)*

**6v) How long did it take to recover from the flu?**

…. (dropdown list seconds/minutes/hours/days)

**6w) Can you indicate how long after the vaccination you started to cough?**

…. (dropdown list seconds/minutes/hours/days)

**6x) Have you recovered from the cough?**

○ No

○ Yes *(fill in question 6y)*

**6y) How long did it take to recover from the cough?**

…. (dropdown list seconds/minutes/hours/days)

**6z) Can you indicate how long after the vaccination the dyspnea started?**

…. (dropdown list seconds/minutes/hours/days)

**6aa) Have you recovered from the dyspnea?**

○ No

○ Yes *(fill in question 6ab)*

**6ab) How long did it take to recover from the dyspnea?**

…. (dropdown list seconds/minutes/hours/days)

**6ac) Can you indicate how long after the vaccination you started to feel fatigue?**

…. (dropdown list seconds/minutes/hours/days)

**6ad) Have you recovered from the fatigue?**

○ No

○ Yes *(fill in question 6ae)*

**6ae) How long did it take to recover from the fatigue?**

…. (dropdown list seconds/minutes/hours/days)

**6af) Can you indicate how long after the vaccination the sleeping problems started?**

…. (dropdown list seconds/minutes/hours/days)

**6ag) Do you sleep normally again?**

○ No

○ Yes *(fill in question 6h)*

**6ah) How long did it take to sleep normally again?**

…. (dropdown list seconds/minutes/hours/days)

**6ai) Can you indicate how long after the vaccination you started to feel irritable?**

…. (dropdown list seconds/minutes/hours/days)

**6aj) Do you feel normally again?**

○ No

○ Yes *(fill in question 6ak)*

**6ak) How long did it take to feel normally again?**

…. (dropdown list seconds/minutes/hours/days)

**7) Did you experience one of the following complaints within one week after the vaccination?**

No Yes Do not know

Decreased appetite ○ ○ (fill in question 7a to 7c) ○

Nausea ○ ○ (fill in question 7d to 7f) ○

Vomiting ○ ○ (fill in question 7g to 7i) ○

Diarrhea ○ ○ (fill in question 7j to 7l) ○

Lower abdominal pain ○ ○ (fill in question 7m to 7o) ○

Dizziness ○ ○ (fill in question 7p to 7r) ○

Fainting ○ ○ (fill in question 7s to 7u) ○

**7a) Can you indicate how long after the vaccination the decreased appetite started?**

…. (dropdown list seconds/minutes/hours/days)

**7b) Has your appetite returned to normal?**

○ No

○ Yes *(fill in question 7c)*

**7c) How long did it take for your appetite to return to normal?**

…. (dropdown list seconds/minutes/hours/days)

**7d) Can you indicate how long after the vaccination the nausea started?**

…. (dropdown list seconds/minutes/hours/days)

**7e) Have you recovered from the nausea?**

○ No

○ Yes *(fill in question 7f)*

**7f) How long did it take to recover from the nausea?**

…. (dropdown list seconds/minutes/hours/days)

**7g) Can you indicate how long after the vaccination did you start vomiting?**

…. (dropdown list seconds/minutes/hours/days)

**7h) Have you recovered from vomiting?**

○ No

○ Yes *(fill in question 7i)*

**7i) How long did it take to recover from vomiting?**

…. (dropdown list seconds/minutes/hours/days)

**7j) Can you indicate how long after the vaccination the diarrhea started?**

…. (dropdown list seconds/minutes/hours/days)

**7k) Have you recovered from the diarrhea?**

○ No

○ Yes *(fill in question 7l)*

**7l) How long did it take to recover from the diarrhea?**

…. (dropdown list seconds/minutes/hours/days)

**7m) Can you indicate how long after the vaccination the lower back pain started?**

…. (dropdown list seconds/minutes/hours/days)

**7n) Have you recovered from the lower back pain?**

○ No

○ Yes *(fill in question 7o)*

**7o) How long did it take to recover from the lower back pain?**

…. (dropdown list seconds/minutes/hours/days)

**7p) Can you indicate how long after the vaccination the dizziness started?**

…. (dropdown list seconds/minutes/hours/days)

**7q) Have you recovered from the dizziness?**

○ No

○ Yes *(fill in question 7r)*

**7r) How long did it take to recover from the dizziness?**

…. (dropdown list seconds/minutes/hours/days)

**7s) Can you indicate how long after the vaccination you fainted?**

…. (dropdown list seconds/minutes/hours/days)

**7t) Have you recovered from the fainting?**

○ No

○ Yes *(fill in question 7u)*

**7u) How long did it take to recover from the fainting?**

…. (dropdown list seconds/minutes/hours/days)

**8) And did you experience one of the following complaints within the week after the vaccination?**

No Yes Do not know

Myalgia ○ ○ (fill in question 8a to 8c) ○

Joint pain ○ ○ (fill in question 8d to 8f) ○

Muscular spasm ○ ○ (fill in question 8g to 8i) ○

Transpire ○ ○ (fill in question 8j to 8l) ○

Rash ○ ○ (fill in question 8m to 8o) ○

Itch ○ ○ (fill in question 8p to 8r) ○

**8a) Can you indicate how long after the vaccination the myalgia started?**

…. (dropdown list seconds/minutes/hours/days)

**8b) Have you recovered from the myalgia?**

○ No

○ Yes *(fill in question 8c)*

**8c) How long did it take to recover from the myalgia?**

…. (dropdown list seconds/minutes/hours/days)

**8d) Can you indicate how long after the vaccination the pain in your joint(s) started?**

…. (dropdown list seconds/minutes/hours/days)

**8e) Have you recovered from the pain in your joint(s)?**

○ No

○ Yes *(fill in question 8f)*

**8f) How long did it take to recover from pain in your joint(s)?**

…. (dropdown list seconds/minutes/hours/days)

**8g) Can you indicate how long after the vaccination the muscular spasm started?**

…. (dropdown list seconds/minutes/hours/days)

**8h) Have you recovered from the muscular spasm?**

○ No

○ Yes *(fill in question 8i)*

**8i) How long did it take to recover from the muscular spasm?**

…. (dropdown list seconds/minutes/hours/days)

**8j) Can you indicate how long after the vaccination you started to transpire more than normal?**

…. (dropdown list seconds/minutes/hours/days)

**8k) Has the level of transpiration returned to normal?**

○ No

○ Yes *(fill in question 8l)*

**8l) How long did it take that the level of transpiration was returned to normal?**

…. (dropdown list seconds/minutes/hours/days)

**8m) Can you indicate how long after the vaccination the rash occurred?**

…. (dropdown list seconds/minutes/hours/days)

**8n) Have you recovered from the rash?**

○ No

○ Yes *(fill in question 8o)*

**8o) How long did it take to recover from the rash?**

…. (dropdown list seconds/minutes/hours/days)

**8p) Can you indicate how long after the vaccination the itch started?**

…. (dropdown list seconds/minutes/hours/days)

**8q) Have you recovered from the itch?**

○ No

○ Yes *(fill in question 8r)*

**8r) How long did it take to recover from the itch?**

…. (dropdown list seconds/minutes/hours/days)

**9) Have you had any other complaint(s) within the week after the vaccination that have not been mentioned above?**

○ No

○ Yes *(fill in question 9a to 9d)*

**9a) What other complaint(s) did you experience?**

**………**

**(question 9b to 9d will be asked for each complaint)**

**9b) Can you indicate how long after the vaccination the {complaint} started ?**

…. (dropdown list seconds/minutes/hours/days)

**9c) Have you recovered from this {complaint}?**

○ No

○ Yes *(fill in question 9d)*

**9d) How long did it take to recover from this {complaint}?**

…. (dropdown list seconds/minutes/hours/days)

***Complete questions 10 to 13 only if you experienced a complaint within the week after the vaccination***

**10) Have you been reported absent from school, sports and/or other activities within the week after the vaccination related to the complaints that have occurred?**

No Yes

School ○ ○ (fill in question 10a)

Sport ○ ○ (fill in question 10b)

Other activities ○ ○ (fill in question 10c and d)

**10a) How long have you been absent from school?**

○ less than 1 day

○ 1 day

○ 2 days

○ 3 days

○ 4 days

○ 5 days

○ 6 days

○ 7 days

**10b) How long have you not been exercising?**

○ less than 1 day

○ 1 day

○ 2 days

○ 3 days

○ 4 days

○ 5 days

○ 6 days

○ 7 days

**10c) What kind of activities were you unable to do?**

……

**10d) How long were you unable to these activities?**

○ less than 1 day

○ 1 day

○ 2 days

○ 3 days

○ 4 days

○ 5 days

○ 6 days

○ 7 days

**11) Have your parents or someone else taken time off from work within the week after the vaccination to take care of you related to the complaints you experienced?**

○ No

○ Yes *(fill in question 11a)*

**11a) How long did he/she take time off from work?**

…. (in hours or days)

**12) Did you take analgesics or other medication within the week after the vaccination related to the complaints you experienced?**

○ No

○ Yes *(fill in question 12a and 12b)*

**12a) Which medicines did you use to treat these complaints?**

**…….**

**12b) How long did you use these medicines(s)?**

…. (dropdown list hours/days)

**13) Did you need medical help within the week after the vaccination related to the complaints you experienced?**

○ No

○ Yes *(fill in question 13a to 13c)*

**13a) What kind of medical help has been sought related to the complaints you experienced?**

□ Contact youth health care organization by phone

□ Contact general practitioner by phone

□ Visit general practitioner

□ Visit First Aid in hospital

□ Visit medical doctor in hospital

□ Admission to hospital

□ Other, namely: **…….**

**13b) Can you describe the complaints for which you sought medical help?**

…..

**13c) How long after the vaccination did you seek medical help?**

…. (dropdown list hours/days)

***This is the end of this questionnaire. Thank you for your participation!***

Send
