# Supplementary material for: Tolerability of MenACWY-TT vaccination in adolescents in the Netherlands; a cross-sectional study
Source: BMC Public Health. 2021 Sep 26;21:1752. doi: 10.1186/s12889-021-11767-9 (PMC8474790; doi:10.1186/s12889-021-11767-9)
Supplement: Supplementary file 3 — Additional file 3. Questionnaire about unrecovered complaints 2 weeks after the MenACWY vaccination. [file 12889_2021_11767_MOESM3_ESM.docx]

**Questionnaire about unrecovered complaints two weeks after the MenACWY vaccination**

Two weeks ago you received the vaccination that protects against four forms of meningococcal disease (MenACWY). The questionnaire below is about complaints that you experienced after the vaccination and that were not recovered one week after the vaccination. Completing this list will take approximately 10 to 15 minutes.

**Notes for completing the questionnaire:**

Different types of questions are asked in the questionnaire. For questions with square boxes you can give multiple answers, in a round box only one answer is possible.

When you have answered the questions, you can click on 'send'.

*One or more of the following questions are only displayed if in the previous questionnaire (i.e. 1 week after the vaccination) the answer was no to the question whether the complaint had recovered.*

**The following question are related to injection site reactions**

**1) Is the swelling around the injection site recovered?**

○ No

○ Yes (go to question 1a)

**1a) How long did it take to recover from the swelling?**

…. (dropdown list minutes/hours/days)

**2) Is the redness around the injection site recovered?**

○ No

○ Yes (go to question 2a)

**2a) How long did it take to recover from the redness?**

…. (dropdown list minutes/hours/days)

**3) Is the pain around the injection site recovered?**

○ No

○ Yes (go to question 3a)

**3a) How long did it take to recover from the pain?**

…. (dropdown list minutes/hours/days)

**4) Is the swelling of the armpit recovered?**

○ No

○ Yes (go to question 4a)

**4a) How long did it take to recover from the swelling of the armpit?**

…. (dropdown list minutes/hours/days)

**5) Can you use your arm normally again?**

○ No

○ Yes *(fill in question 5a)*

**5a) How long did it take that you could use your arm normally again?**

…. (dropdown list seconds/minutes/hours/days)

***The following questions are related to general complaints***

**6) Have you recovered from the listlessness?**

○ No

○ Yes *(fill in question 6a)*

**6a) How long did it take to recover from the listlessness?**

…. (dropdown list seconds/minutes/hours/days)

**7) Have you recovered from the cold?**

○ No

○ Yes *(fill in question 7a)*

**7a) How long did it take to recover from the cold?**

…. (dropdown list seconds/minutes/hours/days)

**8) Have you recovered from the fever**

○ No

○ Yes *(fill in question 8a)*

**8a) How long did it take to recover from the fever?**

…. (dropdown list seconds/minutes/hours/days)

**9) Have you recovered from the headache**

○ No

○ Yes *(fill in question 9a)*

**9a) How long did it take to recover from the headache?**

…. (dropdown list seconds/minutes/hours/days)

**10) Have you recovered from the flu?**

○ No

○ Yes *(fill in question 10a)*

**10a) How long did it take to recover from the flu?**

…. (dropdown list seconds/minutes/hours/days)

**11) Have you recovered from the cough?**

○ No

○ Yes *(fill in question 11a)*

**11a) How long did it take to recover from the cough?**

…. (dropdown list seconds/minutes/hours/days)

**12) Have you recovered from the dyspnea?**

○ No

○ Yes *(fill in question 12a)*

**12a) How long did it take to recover from the dyspnea?**

…. (dropdown list seconds/minutes/hours/days)

**13) Have you recovered from the fatigue?**

○ No

○ Yes *(fill in question 13a)*

**13a) How long did it take to recover from the fatigue?**

…. (dropdown list seconds/minutes/hours/days)

**14) Do you sleep normally again?**

○ No

○ Yes *(fill in question 14a)*

**14a) How long did it take to sleep normally again?**

…. (dropdown list seconds/minutes/hours/days)

**15) Is your increased irritation level back to normal?**

○ No

○ Yes *(fill in question 15a)*

**15a) How long did it take to feel normal again?**

…. (dropdown list seconds/minutes/hours/days)

**16) Has your appetite returned to normal?**

○ No

○ Yes *(fill in question 16a)*

**16a) How long did it take for your appetite to return to normal?**

…. (dropdown list seconds/minutes/hours/days)

**17) Have you recovered from the nausea?**

○ No

○ Yes *(fill in question 17a)*

**17a) How long did it take to recover from the nausea?**

…. (dropdown list seconds/minutes/hours/days)

**18) Have you recovered from vomiting?**

○ No

○ Yes *(fill in question 18a)*

**18a) How long did it take to recover from vomiting?**

…. (dropdown list seconds/minutes/hours/days)

**19) Have you recovered from the diarrhea?**

○ No

○ Yes *(fill in question 19a)*

**19a) How long did it take to recover from the diarrhea?**

…. (dropdown list seconds/minutes/hours/days)

**20) Have you recovered from the lower back pain?**

○ No

○ Yes *(fill in question 20a)*

**20a) How long did it take to recover from the lower back pain?**

…. (dropdown list seconds/minutes/hours/days)

**21) Have you recovered from the dizziness?**

○ No

○ Yes *(fill in question 21a)*

**21a) How long did it take to recover from the dizziness?**

…. (dropdown list seconds/minutes/hours/days)

**22) Have you recovered from the fainting?**

○ No

○ Yes *(fill in question 22a)*

**22a) How long did it take to recover from the fainting?**

…. (dropdown list seconds/minutes/hours/days)

**23) Have you recovered from the myalgia?**

○ No

○ Yes *(fill in question 23a)*

**23a) How long did it take to recover from the myalgia?**

…. (dropdown list seconds/minutes/hours/days)

**24) Have you recovered from the pain in your joint(s)?**

○ No

○ Yes *(fill in question 24a)*

**24a) How long did it take to recover from pain in your joint(s)?**

…. (dropdown list seconds/minutes/hours/days)

**25) Have you recovered from the muscular spasm?**

○ No

○ Yes *(fill in question 25a)*

**25a) How long did it take to recover from the muscular spasm?**

…. (dropdown list seconds/minutes/hours/days)

**26) Has the level of transpiration returned to normal?**

○ No

○ Yes *(fill in question 26a)*

**26a) How long did it take that the level of transpiration was returned to normal?**

…. (dropdown list seconds/minutes/hours/days)

**27) Have you recovered from the rash?**

○ No

○ Yes *(fill in question 27a)*

**27a) How long did it take to recover from the rash?**

…. (dropdown list seconds/minutes/hours/days)

**28) Have you recovered from the itch?**

○ No

○ Yes *(fill in question 28a)*

**28a) How long did it take to recover from the itch?**

…. (dropdown list seconds/minutes/hours/days)

***The following question are related to other complaints you mentioned previously {repeated section}***

**29) Have you recovered from this {complaint}?**

○ No

○ Yes *(fill in question 29a)*

**29a) How long did it take to recover from this {complaint}?**

…. (dropdown list seconds/minutes/hours/days)

**Attention! The following questions are about the period after completing the previous questionnaire.**

**30) Have you been reported absent from school, sports and/or other activities in the past week related to the complaints that have occurred?**

No Yes

School ○ ○ (fill in question 30a)

Sport ○ ○ (fill in question 30b)

Other activities ○ ○ (fill in question 30c and d)

**30a) How long have you been absent from school?**

○ less than 1 day

○ 1 day

○ 2 days

○ 3 days

○ 4 days

○ 5 days

○ 6 days

○ 7 days

**30b) How long have you not been exercising?**

○ less than 1 day

○ 1 day

○ 2 days

○ 3 days

○ 4 days

○ 5 days

○ 6 days

○ 7 days

**30c) What kind of activities were you unable to do?**

……

**30d) How long were you unable to these activities?**

○ less than 1 day

○ 1 day

○ 2 days

○ 3 days

○ 4 days

○ 5 days

○ 6 days

○ 7 days

**31) Have your parents or someone else taken time off from work in the past week to take care of you related to the complaints you experienced?**

○ No

○ Yes *(fill in question 31a)*

**31a) How long did he/she take time off from work?**

…. (in hours or days)

**32) Did you take analgesics or other medication in the past week related to the complaints you experienced?**

○ No

○ Yes *(fill in question 32a and 32b)*

**32a) Which medicines did you use to treat these complaints?**

**…….**

**32b) How long did you use these medicines(s)?**

…. (dropdown list hours/days)

**33) Did you need medical help in the past week related to the complaints you experienced?**

○ No

○ Yes *(fill in question 33a to 33c)*

**33a) What kind of medical help has been sought related to the complaints you experienced?**

□ Contact youth health care organization by phone

□ Contact general practitioner by phone

□ Visit general practitioner

□ Visit First Aid in hospital

□ Visit medical doctor in hospital

□ Admission to hospital

□ Other, namely: **…….**

**33b) Can you describe the complaints for which you sought medical help?**

…..

**33c) How long after the vaccination did you seek medical help?**

…. (dropdown list hours/days)

***This is the end of this questionnaire. Thank you for your participation!***

Send
